# Validation of bioelectrical impedance analysis in Ethiopian adults with HIV

**DOI:** 10.1017/jns.2017.67

**Published:** 2017-12-18

**Authors:** Maria H. Hegelund, Jonathan C. Wells, Tsinuel Girma, Daniel Faurholt-Jepsen, Dilnesaw Zerfu, Dirk L. Christensen, Henrik Friis, Mette F. Olsen

**Affiliations:** 1 Department of Public Health, University of Copenhagen, Copenhagen, Denmark; 2 Department of Nutrition, Exercise and Sports, University of Copenhagen, Copenhagen, Denmark; 3 Childhood Nutrition Research Centre, Great Ormond Street Institute of Child Health, University College London, London, UK; 4 Department of Paediatrics and Child Health, Jimma University, Jimma, Ethiopia; 5 Department of Infectious Diseases, Rigshospitalet, Copenhagen, Denmark; 6 Ethiopian Public Health Institute, Addis Ababa, Ethiopia

**Keywords:** Bioelectrical impedance analysis, Body composition, HIV, African population, ART, antiretroviral therapy, BIA, bioelectrical impedance analysis, FFM, fat-free mass, FM, fat mass

## Abstract

Bioelectrical impedance analysis (BIA) is an inexpensive, quick and non-invasive method to determine body composition. Equations used in BIA are typically derived in healthy individuals of European descent. BIA is specific to health status and ethnicity and may therefore provide inaccurate results in populations of different ethnic origin and health status. The aim of the present study was to test the validity of BIA in Ethiopian antiretroviral-naive HIV patients.

BIA was validated against the ^2^H dilution technique by comparing fat-free mass (FFM) measured by the two methods using paired *t* tests and Bland–Altman plots. BIA was based on single frequency (50 kHz) whole-body measurements. Data were obtained at three health facilities in Jimma Zone, Oromia Region, South-West Ethiopia. Data from 281 HIV-infected participants were available. Two-thirds were female and the mean age was 32·7 (sd 8·6) years. Also, 46 % were underweight with a BMI below 18·5 kg/m^2^. There were no differences in FFM between the methods. Overall, BIA slightly underestimated FFM by 0·1 kg (−0·1, 95 % CI −0·3, 0·2 kg). The Bland–Altman plot indicated acceptable agreement with an upper limit of agreement of 4·5 kg and a lower limit of agreement of −4·6 kg, but with a small correlation between the mean difference and the average FFM. BIA slightly overestimated FFM at low values compared with the ^2^H dilution technique, while it slightly underestimated FFM at high values. In conclusion, BIA proved to be valid in this population and may therefore be useful for measuring body composition in routine practice in HIV-infected African individuals.

Malnutrition is common in individuals infected with HIV in sub-Saharan Africa. Chronic infections such as HIV result in immune impairment which leads to malnutrition, causing further immune impairment and thereby a more rapid disease progression. On the other hand, malnourished individuals have increased susceptibility to HIV and opportunistic infections, and are thereby more likely to have a faster disease progression compared with well-nourished individuals^(^^1^^)^.

The wasting syndrome in HIV is characterised by weight loss often accompanied by chronic diarrhoea or chronic weakness and fever^(^^2^^)^. It has been one of the main characteristics of HIV and it is still a common complication in the antiretroviral therapy (ART) era^(^^3^^)^. In HIV-infected individuals starting ART, malnutrition may be an independent predictor of early mortality^(^^4^^)^. Measurement of body composition is an important tool to assess effects of weight loss and therefore it is crucial to find an easy, quick and valid method to determine body composition.

Bioelectrical impedance analysis (BIA) is an inexpensive, non-invasive and easy-to-use method to determine body composition^(^^5^^)^. The most widely used approach predicts values for total body water, allowing calculation of fat-free mass (FFM) and fat mass (FM). Total body water predictions are typically calibrated using data from a healthy reference population, often of white European descent, using a reference method such as dual-energy X-ray absorptiometry and regression formulae that include height and impedance as variables, but often also other terms such as age, weight and sex^(^^5^^)^. The hydration of FFM is considered relatively constant through adulthood in healthy individuals^(^^6^^)^, but several illnesses and conditions such as HIV and malnutrition are associated with weight loss and thereby alter body composition^(^^7^^)^, potentially also affecting hydration.

Additionally, the manufacturers’ equations used in BIA are typically derived in individuals of European descent^(^^8^^)^. Several validation studies using the ^2^H dilution technique as the reference method have concluded that BIA was a valid method to determine body composition in HIV-infected individuals^(^^9^^,^^10^^)^. One of the studies was conducted in an American cohort and included black Americans^(^^9^^)^. The other study was conducted in Europe based on data from a small sample^(^^10^^)^. However, the accuracy of these equations, when used in African HIV-infected individuals has subsequently been questioned^(^^11^^)^. Therefore, equations used in BIA based on healthy subjects of European descent may provide inaccurate results in Africans with HIV.

The aim of the present study was to test the validity of BIA for the assessment of FFM in ART-naive Ethiopian HIV-infected patients.

## Methods

### Study design and population

This study used baseline data from the ARTfood study^(^^12^^)^, which was a randomised controlled trial investigating the effects and feasibility of providing a lipid-based nutrient supplement in HIV-infected patients at initiation of ART. The sample size was calculated for the primary outcome of the trial.

Participants were recruited among HIV patients eligible for ART and took place at Jimma University Specialised Hospital, and health centres in Jimma and Agaro. The inclusion criteria for the ARTfood study were ≥18 years, BMI ≥16 kg/m^2^, ART-naive, eligible for initiation of ART, and living within 50 km of the recruitment facility. Patients were excluded if they were pregnant, lactating, taking micronutrients or other nutrient supplementation. Patients with BMI <16 kg/m^2^ were invited for data collection and therefore included in this study, whereas they were excluded from intervention and referred to standard nutritional therapy according to national guidelines^(^^13^^)^.

Eligibility of ART during the study was based on the Ethiopian treatment guidelines from 2008. HIV patients were eligible if they had CD4 count ≤200 cells/μl irrespective of clinical symptoms, CD4 count ≤350 cells/μl if WHO stage III, or WHO stage IV irrespective of CD4 count^(^^14^^)^.

### Data collection

The study staff included nurses, laboratory technicians and pharmacists, all receiving relevant training. Data collection was carried out from July 2010 to July 2013.

### Background data

Background data were collected through structured questionnaires in the local languages Amharic or Afaan Oromo. For the present study, data on age, sex, education and occupation were used.

### Anthropometric data

For height and weight measurements, participants were barefoot and wearing light clothes. A calibrated stadiometer (SECA 214 Stadiometer) and scale (Tanita-BC 418 MA) were used for height and weight, respectively. Weight was measured with 0·1 kg precision and height to the nearest 1 mm. BMI was calculated as weight divided by squared height (kg/m^2^) and categorised as <16·0, 16·0–<17·0, 17·0–<18·5, 18·5–25·0 or >25·0 kg/m^2^ according to WHO classification^(^^15^^)^.

### HIV status

Information regarding clinical stage of HIV using WHO criteria^(^^16^^)^ was extracted from patient records and checked by a study clinician. CD4 cell count was determined in EDTA-stabilised whole blood using flow cytometry (Fascount; Becton Dickinson) and categorised into <50, 50–<100, 100–200 and >200 cells/μl for analyses. To determine viral load, plasma was kept at −80°C before quantification of HIV-1 viral load using a commercial real-time PCR assay (RealTimeHIV-1; Abbott Laboratories) with automated extraction (M2000 Real Time System, Abbott Laboratories). HIV viral load was categorised as <4, 4–5 and >5 log (1 + copies/ml)^(^^17^^)^.

### ^2^H dilution technique

Body composition was assessed with 30 g ^2^H-labelled water (99·8 % ^2^H; Sercon) weighed with 0·01 g precision and given orally after collection of pre-dose saliva samples. Post-dose saliva samples were collected after 4 h equilibration^(^^18^^)^. Saliva enrichment of ^2^H was determined by a Fourier transform infrared spectrometer (IRAffinity-1; Shimadzu). Total body water was calculated from post-dose ^2^H enrichment with adjustment for pre-dose enrichment, using a factor of 1·041 to adjust for proton exchange. FFM was calculated based on an assumed hydration factor of 73·2 %^(^^18^^)^. FM was thereafter calculated by subtracting FFM from total body weight^(^^18^^)^.

### Bioelectrical impedance analysis

Body composition was also measured using single-frequency eight-electrode BIA (Tanita-BC 418 MA). Participants were barefoot, wearing light clothes and asked to empty pockets and remove any metal objects. The Tanita body composition analyser measures body composition using a constant current source at a frequency of 50 kHz and provides impedance (*Z*) measured in ohms. Fat percentage, FM and FFM are produced by regression algorithms generated by the manufacturer, the details of which are not available, though it is described that the algorithms are based on data from ‘Western’ and Japanese individuals^(^^19^^)^. BIA assessment was available for all participants recruited at Jimma University Specialised Hospital and the health centre in Jimma, but for logistic reasons only for some of the participants attending the health centre in Agaro.

### Data analysis

The collected data were double-entered and validated using Epidata (EpiData Association, Denmark). Data analyses were carried out using STATA/IC version 13.0 (StataCorp LLC). FFM was used as the primary outcome to compare BIA against the ^2^H dilution technique. Paired *t* tests were used to compare FFM measured by the two different methods. Overall two paired *t* tests were conducted. The first test was stratified by sex and age group (18–<30, 30–<40 and >40 years). The second test was stratified by CD4 cell count including <50, 50–<100, 100–200 and >200 cells/μl. Values in the tables are mean values and standard deviations for FFM and mean differences (95 % CI). *P* values <0·05 were considered significant.

The Bland–Altman plot was used to evaluate agreement between FFM measured by BIA and the ^2^H dilution technique. Furthermore, regression models were conducted for FFM and FM to test if there were correlations between the mean difference and average by the two methods.

Additionally, a calibration equation was generated from our own data. The participants were equally divided into two random samples using a random sampling generator (STATA/IC version 13; StataCorp LLC). One sample was used to develop the equation through multiple linear regression for prediction of FFM as measured by the ^2^H dilution technique. The multiple regression model included impedance index (HT^2^/z), weight, age and sex as predictors. The other sample was used to test the predictive ability of the equation using limits of agreement. The predicted FFM was tested against FFM as measured by BIA and the ^2^H dilution technique, respectively.

## Results

Of 453 HIV-infected patients screened between July 2010 and August 2012, 348 (77 %) were recruited for the ARTfood study. Participants were younger (32·9 *v.* 37·0 years; *P* = 0·001) and had lower education (21 *v.* 45 % with secondary school or higher; *P* < 0·001) than those not recruited, while BMI and other demographic characteristics were similar^(^^20^^)^. The ^2^H dilution technique was used for the primary outcome of the trial and the Tanita body composition analyser was not available at all sites. Of the recruited participants, data from both BIA and the ^2^H dilution technique were available in 281 (81 %) participants and therefore included in the present validation study.

Characteristics of the 281 HIV-infected participants are shown in Table 1. Two-thirds (68 %) of the participants were female and the mean age was 32·7 (sd 8·6) years. There was a high prevalence of underweight, as almost half (46 %) of the participants had BMI <18·5 kg/m^2^.

**Table 1.** Characteristics of antiretroviral therapy-naive individuals infected with HIV (Numbers of subjects and percentages or mean values and standard deviations)

	HIV positive (*n* 281)	
	*n*	%
Background characteristics		
Age (years)		
Mean	32·7	
sd	8·6	
Female	190	67·6
Education		
No formal schooling*	70	24·9
Primary†	144	51·3
Secondary or higher‡	67	23·8
Occupation		
Small-scale trader/merchant	32	11·4
Formal job sector public/private	68	24·2
Informal daily labourer/farmer, etc.	79	28·1
Unemployed/housewife	87	31·0
Other	15	5·3
Anthropometric characteristics		
Height (cm)		
Mean	160·4		
sd	8·3		
Weight (kg)		
Mean	49·3		
sd	7·9		
BMI (kg/m^2^)§		
<16	23	8·2
16·0–16·9	30	10·7
17·0–18·49	76	27·1
18·5–24·9	144	51·3
>25	8	2·9
Fat-free mass (kg)‖		
Mean	39·5		
sd	6·4		
Fat mass (kg)‖		
Mean	9·7		
sd	5·0		
HIV status		
CD4 count (cells/μl)		
<50	18	6·6
50–100	36	13·2
100–200	101	37·0
>200	118	43·2
Viral load, log (1 + copies/ml)		
<4	45	16·5
4–5	112	41·0
>5	116	42·5
WHO stage		
Stage I	80	29·0
Stage II	78	28·3
Stage III	92	33·3
Stage IV	26	9·4

* No formal schooling/only able to read and write.

† Some primary school/finished primary school.

‡ Finished secondary school/attended higher education.

§ BMI was classified according to the WHO classification, which is the International Classification of adult underweight, overweight and obesity according to BMI^(^^15^^)^.

‖ Measured using the ^2^H dilution technique.

Mean CD4 count was 196·2 (sd 116) cells/μl and 57 % of the participants had ≤ 200 CD4 cells/μl. More than 40 % had viral load >5 log (1 + copies/ml) and more than two-thirds (71 %) were symptomatic (i.e. in WHO clinical stage II, III or IV).

There were no significant differences between the two methods. The overall mean FFM measured by BIA was 39·5 kg, whereas the overall mean FFM measured by the ^2^H dilution technique was 39·6 kg. The mean difference between these two methods was −0·1 (95 % CI −0·3, 0·2) kg. For males, the mean FFM was 46·2 kg when assessed by BIA and 46·3 kg when assessed by the ^2^H dilution technique with a mean difference of −0·2 (95 % CI −0·7, 0·4) kg (Table 2). For females, the mean FFM was 36·3 kg when assessed by BIA and 36·4 kg when assessed by the ^2^H dilution technique with a mean difference of −0·0 (95 % CI −0·3, 0·3) kg. Among those with CD4 count <50 cells/μl, the mean difference was 1·3 kg (*P* = 0·06) (Table 3).

**Table 2.** Comparison of fat-free mass measured through the ^2^H dilution technique *v.* bioelectrical impedance analysis (BIA) in HIV-infected individuals stratified by age group and sex* (Mean values and standard deviations)

		Fat-free mass BIA		Fat-free mass ^2^H dilution				
Age group	Participants (*n*)	Mean	sd	Mean	sd	Difference	95 % CI	*P*
Males†	92	46·2	5·2	46·3	5·1	−0·2	−0·7, 0·4	0·58
18–29 years	14	45·0	5·6	44·9	5·4	0·2	−1·2, 1·6	0·77
30–39 years	43	47·8	5·4	47·5	5·5	0·3	−0·4, 1·0	0·46
>40 years	35	44·6	4·5	45·4	4·3	−0·8	−2·0, 0·3	0·16
Females‡	189	36·3	3·1	36·4	4·0	−0·0	−0·3, 0·3	0·82
18–29 years	94	36·5	2·9	36·5	4·1	−0·0	−0·4, 0·4	0·97
30–39 years	71	36·2	2·9	36·4	3·6	−0·2	−0·7, 0·2	0·37
>40 years	24	36·1	4·2	35·7	4·9	0·4	−0·5, 1·3	0·38

* Fat-free mass measured through the ^2^H dilution technique and BIA by the Tanita body composition analyser was compared using paired *t* tests.

† All HIV-infected male participants with BIA and ^2^H dilution data.

‡ All HIV-infected female participants with BIA and ^2^H dilution data.

**Table 3.** Comparison of fat-free mass from the ^2^H dilution technique and fat-free mass measured using bioelectrical impedance analysis (BIA) in HIV-infected individuals stratified by CD4 cell count* (Mean values and standard deviations)

		Fat-free mass BIA		Fat-free mass ^2^H dilution				
CD4 cell count	Participants (*n*)	Mean	sd	Mean	sd	Difference	95 % CI	*P*
All†	281	39·5	6·1	39·6	6·4	−0·1	−0·3, 0·2	0·58
<50 cells/μl	18	39·9	6·5	38·6	5·7	1·3	−0·0, 2·6	0·06
50–100 cells/μl	37	39·8	6·0	40·2	6·3	−0·4	−1·1, 0·2	0·19
100–200 cells/μl	103	39·6	6·0	39·8	6·7	−0·1	−0·6, 0·3	0·52
>200 cells/μl	123	39·4	6·1	39·5	6·4	−0·1	−0·5, 0·3	0·62

* Fat-free mass measured through the ^2^H dilution technique and BIA by the Tanita body composition analyser was compared using paired *t* tests.

† All HIV-infected participants with BIA and ^2^H data.

**Fig. 1.**
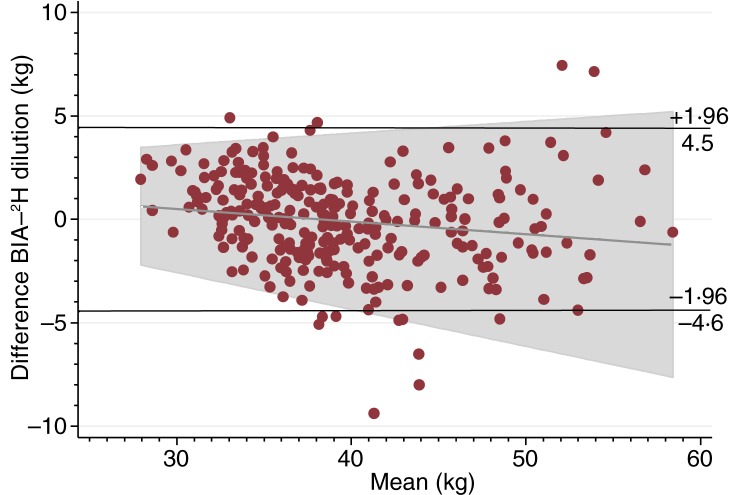
Bland–Altman plot including regression line of difference *v.* mean, comparing fat-free mass measured by the ^2^H dilution technique and bioelectrical impedance analysis (BIA).

Fig. 1 shows the Bland–Altman plot of FFM measured by the ^2^H dilution technique and BIA using Tanita including the regression line of the mean difference and average for FFM. BIA underestimated the ^2^H dilution technique with a mean difference of −0·1 (sd 2·3) kg between the two methods. These data correspond to an upper limit of agreement of 4·5 kg and a lower limit of agreement of −4·6 kg. Therefore, since the differences between the methods were normally distributed (data not shown), 95 % of the differences are expected to lie between −4·6 and 4·5 kg. The regression model for FFM showed a small correlation of −0·1 (se 0·02) (*P* = 0·01) between the mean difference and the average and with an *R*^2^ of 0·02. For an average FFM of 28 kg, the regression line predicted a mean difference of 0·6 kg, whereas the predicted mean difference was −1·2 kg for an average FFM of 58 kg. There was no correlation (*P* > 0·05) between the mean difference and the average for FM (data not shown).

The total sample (*n* 281) was randomised into two subsamples. One was used to develop a predictive equation (equation sample) and the other to validate the equation (validation sample). There were no significant differences in characteristics between the two subsamples (data not shown). Based on data from the equation sample, FFM by the ^2^H dilution technique was predicted by the following equation:

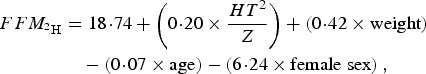

where height (*HT*) is in cm, *Z* is in ohms, weight is in kg, age is in years, and female sex is a dummy variable (*R*^2^ 0·82; standard error of estimate 2·6 kg).

The predicted FFM was tested in the validation sample against Tanita and resulted in a mean difference of −0·1 (sd 2·2) kg and an upper limit of agreement of 4·3 kg and a lower limit of agreement of −4·5 kg. The predicted FFM tested against the ^2^H dilution technique resulted in a mean difference of −0·1 (sd 2·9) kg and an upper limit of agreement of −5·8 kg and a lower limit of agreement of 5·6 kg.

## Discussion

In this cohort, we compared BIA by the Tanita body composition analyser against the reference method of ^2^H dilution for determining FFM in Ethiopian HIV-infected individuals. There were no differences between FFM measured by the two methods neither in the comparison stratified by age and sex, nor in the comparison stratified by CD4 cell count. In the strata <50 CD4 cells/μl and males aged 18–29 years, there were relatively few participants with *n* 18 in the first and *n* 14 in the latter. These small subgroups may have caused type II error, potentially failing to detect a difference between FFM measured by BIA and the ^2^H dilution technique.

The Bland–Altman plot also indicated acceptable agreement between the two methods with a small mean difference and limits of agreements similar to other BIA studies in adults^(^^11^^,^^21^^)^. However, there was a small correlation between the mean difference and the average FFM with an *R*^2^ value of 0·02 indicating that 2 % of the variance in the mean difference was due to the level of FFM. The regression line indicated that BIA slightly overestimated the ^2^H dilution technique at low FFM, while it underestimated at high FFM values. Nonetheless, these differences are considered minimal.

CD4 cell count is an indicator of health status and disease progression in HIV-infected individuals. Healthy individuals have a CD4 cell count between 500 and 1600 cells/μl^(^^22^^)^. In the present study, approximately 60 % of the HIV-infected participants had a CD4 cell count <200 cells/μl, which is considered very low. In this cohort, very low CD4 cell count did not seem to affect validity of BIA significantly. Further research is needed to validate BIA against the ^2^H dilution technique especially in severely ill HIV-infected individuals with a CD4 cell count <50 cells/μl.

HIV-infected individuals may vary in hydration of FFM during the different stages of the disease, caused either by dehydration or fluid retention which may influence bioelectrical resistance in the body and thereby affect the accuracy of BIA measurements^(^^11^^)^. However, this problem will also affect results of the ^2^H dilution technique, which also assumes constant hydration. Equations for BIA measurement derived and validated by Kotler *et al*. in a cohort of white, black and Hispanic HIV-infected and uninfected individuals^(^^9^^)^ were reported as not valid in another study in an African HIV-infected cohort^(^^11^^)^. Surprisingly, among the fifteen published equations they tested, the two equations they found valid in their HIV-infected cohort were developed in uninfected individuals^(^^11^^)^. This is in accordance with the present study where the manufacturer's equation was developed based on data from uninfected individuals.

When using BIA to measure body composition, besides health status and ethnicity, other considerations need to be taken into account. These include potential intra-individual variability in hydration due to shifts in fluids and electrolytes^(^^23^^)^. Intra-individual variability can be divided into inter-day changes and intra-day fluctuations which cause changes in impedance. Changes in impedance of the trunk are very small, whereas impedance changes in the upper and lower limbs are much bigger. Intra-day variability in water content and distribution is due to consumption of food and physical activity^(^^19^^)^, but could also be due to changes in disease status. Inter-day changes are caused by temporary weight changes, for example caused by dehydration, over-eating and/or -drinking^(^^19^^)^. It is therefore important to register and control for behaviour that may change hydration before using the BIA method^(^^23^^)^.

Ethnicity has also been shown to affect the accuracy of BIA, especially in African populations. The validity of BIA equations regarding ethnicity remains uncertain, as some equations have shown validity, while other equations show under- or over-estimation of BIA measurements against the reference method^(^^24^^)^. This may be due to differences in length of limbs and body composition. For example, African individuals generally have longer legs than European individuals, while Asian individuals are known to have shorter legs^(^^8^^)^. This is relevant in BIA because impedance is unequally distributed across regional anatomy and thereby overestimation of FM may occur if the population generally has longer legs than those used in the equations of the BIA method^(^^8^^)^. However, in this cohort African ethnicity did not seem to affect BIA validity despite the fact that the equations in the BIA were based on data from ‘Western’ and Japanese individuals^(^^19^^)^. A possible explanation is that Ethiopian individuals may have body geometry more similar to the reference population than the general African population. Another possible explanation is that the combination of ‘Western’ and Japanese individuals used as the reference population in the equations used by Tanita resulted in valid measurements.

The overall *t* test and the Bland–Altman plot showed a mean difference of −0·1 (sd 2·3) kg with BIA slightly, but not significantly, underestimating FFM compared with the ^2^H dilution technique. The small correlation detected between mean difference and average FFM by the two methods reveals that there was a negligible difference when using BIA compared with the ^2^H dilution technique in this population. Measuring FFM using the Tanita body composition analyser resulted in an error of ±4·6 kg (1·96 sd limits of agreement) in this population. This accuracy is typical for BIA in adults^(^^21^^,^^24^^)^. Furthermore, a standard error of estimate of 2·3 in women and 3·0 in men has been reported as ‘very good performance’^(^^25^^)^. We therefore consider this accuracy of BIA clinically acceptable.

Using our own data, we could predict FFM from the impedance index, age, weight and sex with an error of ±5·2 kg (1·96 sd limits of agreement). This is only slightly less accurate than published predictions using data from healthy African populations^(^^21^^)^. Since all information is known, it would be valuable to conduct further validation using more parameters. It may be preferable to predict total body water instead of FFM to make it more comparable with other published equations.

The main strength of the present study is that it used the ^2^H dilution technique as the reference method, because it is characterised with high accuracy and precision^(^^26^^)^. Another strength is the large number of participants. It is also a strength that the majority of the participants had poor health status and progressed HIV infection (CD4 count <200 CD4 cells/μl), because they are likely to differentiate more from healthy individuals than HIV-infected individuals with a better health status.

However, it may be a limitation that the sample was not representative of HIV patients. The HIV-infected patients were included at ART initiation, based on criteria of the 2008 guideline, and all of them had a low CD4 count or were symptomatic. Therefore, the variety in disease severity was small. Another limitation is the potential type II error in the strata <50 CD4 cells/μl and males aged 18–29 years. The fact that BIA measurements were not available for all participants may also be a limitation. Nonetheless, missing data were due to limitations in logistics and therefore not expected to be associated with outcome. It should be noted that the Tanita body composition analyser validated in this study is a discontinued model and the replaced model uses a different algorithm.

In conclusion, the Tanita body composition analyser is considered a valid tool for the assessment of FFM in this cohort of Ethiopian ART-naive HIV patients. BIA is an easy and inexpensive method to determine body composition and according to the results from this study it was reliable in both males and females in all age groups and also in all CD4 strata. BIA may therefore be a useful method to measure body composition in routine clinical and epidemiological practice in HIV-infected African individuals.
